# Evaluation of method performance for oxidative stress biomarkers in urine and biological variations in urine of patients with type 2 diabetes mellitus and diabetic nephropathy

**DOI:** 10.1186/s12575-015-0015-9

**Published:** 2015-02-02

**Authors:** Ergul Belge Kurutas, Yakup Gumusalan, Ali Cetinkaya, Ekrem Dogan

**Affiliations:** Department of Biochemistry, Sutcu Imam University Faculty of Medicine, Avsar Campus, 46050 Kahramanmaras, Turkey; Department of Anatomy, Fatih University Faculty of Medicine, Büyükçekmece Campus, 34500 Istanbul, Turkey; Department of Gastroenterology, Sutcu Imam University Faculty of Medicine, Kahramanmaras, Turkey; Department of Nephrology, Selahaddin Eyyubi University Faculty of Medicine, Bağlar Campus, 21090 Diyarbakir, Turkey

**Keywords:** Biological variation, DN, Oxidative stress biomarkers, Reference change value, T2DM

## Abstract

**Background:**

Oxidative stress biomarkers such as superoxide dismutase (CuZnSOD), catalase (CAT) and malondialdehyde (MDA) play an important role in the pathogenesis or progression of numerous diseases. Data regarding the biological variation and analytical quality specifications (imprecision, bias and total error) for judging the acceptability of method performance for oxidative stress biomarkers in urine are conspicuously lacking in the literature. Such data are important in setting analytical quality specifications, assessing the utility of population reference intervals (index of individuality) and assessing the significance of changes in serial results from an individual (reference change value; RCV).

**Materials and methods:**

20 patients with type 2 diabetes mellitus (T2DM), 20 patients with diabetic nephropathy (DN) and 14 healthy individuals as control were involved in this study. Timed first morning urine samples were taken from patients and healthy groups on the zero, 1st, 3rd, 5th, 7th, 15th and 30th days. Index of individuality and reference change value were calculated from within-subject and between-subject variations. Methods of oxidative stress biomarkers in human blood were adopted in human urine and markers were measured as spectrophotometrically. Also, analytical quality specifications for evaluation of the method performance were established for oxidative stress biomarkers in urine.

**Results:**

Within-subject variations of oxidative stress biomarkers were significantly higher in patients with DN and T2DM compared to healthy subjects. MDA showed low individuality, and within-subject variances of MDA were larger than between-subject variances in all groups. However, CAT and CuZnSOD showed strong individuality, but within-subject variances of them were smaller than between-subject variances in all groups. RCVs of all analytes in diabetic patients were relatively higher, because of high within-subject variation, resulting in a higher RCV. Also, the described methodology achieves these goals, with analytical CVs of < 3.5% for all analytes. Goals for bias and total error were 6.0-7.9% and 12.5-23.3%, respectively.

**Conclusions:**

RCVs concept for predicting the clinical status in diabetic patients represents an optimization of laboratory reporting and could be a valuable tool for clinical decision. Furthermore, for oxidative stress biomarkers’ measurements in urine, the desirable imprecision goals based on biological variation are obtainable by current methodologies.

## Introduction

Oxidative stress is an imbalance between reactive oxygen species (ROS) and protective radical scavenging antioxidants resulting from either an overproduction of ROS or a deficit in antioxidant protection [1]. Oxidative stress biomarkers such as catalase (CAT), superoxide dismutase (CuZnSOD) and malondialdehyde (MDA) are increasingly being evaluated in experimental, clinical and epidemiological studies and have been implicated in the pathogenesis of numerous diseases including atherosclerosis, cancer, diabetes, respiratory disease and others [2].

The biological component is characterized by the biological within-subject variation during the stable phase of a disease. Many authors have studied within-individual variation in the concentrations of biochemical analytes in serum, plasma and urine from apparently healthy individuals [3-5]. However, one cannot apply data from healthy individuals because the disease itself may influence the magnitude of variation. Using biological variation to derive analytical performance criteria begins with determining how much natural variation is expected for a test result. The concentration of measurand in individuals varies around their baseline or homeostatic set-point. The total variation observed is composed of preanalytical variation, analytical variation (imprecision and bias), and inherent within-person biological variation. In a laboratory setting, we can control preanalytical and analytical variability, leaving the biological component as the only true variable. If preanalytical error related to phlebotomy, transport, handling, and storage of samples is minimized, and the analytical variation is considerably less than the biological variability, serial testing can be used to determine whether a patient has improved [6,7]. Determining the effect of analytical variation relative to biological variation is a straightforward way to develop desirable performance criteria of an assay [8,9].

Measuring the degree of oxidative stress is not in wide clinical use, since no standardized method has been accepted for measuring the oxidative stress status and lipid peroxidation in humans. These analyte measurements are important and the need to investigate relationships between clinical outcome and laboratory tests requires reliable reference values. Some information is available on the biological variations of oxidative stress biomarkers. Browne et al. reported that biologic within-subject variations of oxidative stress biomarkers in erythrocyte didn’t change during menstrual cycle [10]. Diaz et al. showed that oxidative stress biomarkers in human plasma can be adapted for screening patients who may be subject to oxidative stress, and can be used for the routine monitoring of lipid peroxidation in human disorders [11]. We previously showed the levels of oxidative stress biomarkers in urines of patients with urinary tract infection and diabetic patients [12,13]. However, to the best of our knowledge, none of the previous researches investigated the biological variations of antioxidant enzymes (CAT, CuZnSOD) and MDA in urines of healthy individuals as well as in urines of patients with diabetic nephropathy (DN) and type 2 diabetes mellitus (T2DM). Therefore, 1) to assess the components of variation of CAT, CuZnSOD and MDA analytes significantly affected by a pathological process, we investigated the biological variation of CAT, CuZnSOD and MDA analytes in patients with DN and T2DM. 2) we have defined the contributions to the overall variance of analytical, within-, between-subject variance for each of oxidative stress biomarkers in the assessment of DN and T2DM. Furthermore, the data were used to calculate the reference change value (RCV) required for the assessment of the significance of changes in serial results from an individual and to define analytical quality specifications for evaluation of method performance such as imprecision (I), bias (B) and total error (TE).

## Materials and methods

### Subjects

All subjects were volunteers who were informed about the objective of the study before- hand. During the course of the study no illness or injury was observed in the healthy individuals and no additional illness was observed in T2DM and DN patients. American Diabetes Association criteria were used for diagnosis of T2DM [14] and the Kidney Disease Outcomes Quality Initiative criteria were used for diagnosis of DN [15]. During the study, treatment of T2DM or DN patients was not changed, the disease process being considered stable. T2DM and DN patients underwent either a fundoscopic examination or fluoroangiographic study for diagnosis of retinopathy. Patients with drug induced nephrotoxic damage or secondary causes of renal insufficiency such as obstructive renal disease, renal stone disease, and acute urinary tract infection were excluded. The patients who were known to have a familial disease such as autosomal dominant polycystic kidney disease or Alport disease were not included in the study.

#### Healthy reference group

This group consisted 8 females, ages 49 to 59 years (mean 53.0 years), and 7 males, ages 49 to 55 years (mean 52.4 years). All were apparently healthy and none was taking any drugs, including oral contraceptives.

#### T2DM reference group

There were 9 females, aged 50 to 55 years (mean 52.2 years) and 11 males, aged 50 to 62 years (mean 55.0 years) in this group.

#### DN reference group

Ten females, aged 45.0 to 61.0 years (mean 53.0 years) and 10 males, aged 49 to 60 years (mean 54.4 years) were included in this group.

The demographic characteristics of reference groups were listed in Table 1. Approval from the Ethics Committee of Kahramanmaras Sutcu Imam University Medical Faculty was taken in accordance with the principles of Declaration of Helsinki and informed consent was obtained from the cases.

**Table 1:** **Comparisons of demographic and laboratory data among T2DM, DN and healthy individuals**

**Variables**	**T2DM**	**DN**	**Healthy individuals**
Age (years)			
Female	52.20 ± 2.16	53.00 ± 6.78	53.00 ± 3.80
Male	55.00 ± 4.47	54.40 ± 3.91	52.40 ± 2.40
BMI (kg/m^2^)	23.20 ± 1.30	22.98 ± 2.32	22.40 ± 1.51
DM duration, years	18.40 ± 2.30	20.40 ± 3.91	None
Hypertension	8/20	17/20	None
Retinopathy	8/20	14/20	None
Uremia	1/20	19/20	None
Microalbuminuria	None	3/20	None
Proteinuria	None	16/20	None
GFR (mL/min/1.73 m^2^)	100.29 ± 13.59*	43.68 ± 10.29*	116.80 ± 12.31*
HbA1c (mmol/mol)	63.5 ± 9.5*	65.3 ± 6.0*	31.8 ± 5.6
FBG (mmol/L)	8.8 ± 0.4*	7.0 ± 0.5*	4.9 ± 0.9
Serum creatinine (micromol/L)			
Female	123.6 ± 6.15	145.5 ± 8.1	77.4 ± 7.4
Male	137.7 ± 9.15**	163.6 ± 24.3**	105.1 ± 6.9**

*Significant differences in patients with T2DM and DN compared to healthy individuals (p < 0.05).

**Significant differences in creatinine levels between male and female subjects in all groups (p < 0.05).

### Analytical techniques

#### Blood samples

The blood samples were taken from each individual on the zero day of our study. The analytical methods for routine biochemical analysis were standard for the medical laboratories. In brief, the basic principles and instruments involved were as follows. Measurements of total protein (biuret reaction), fasting blood glucose (FBG) (hexokinase/glucose-6-phosphate dehydrogenase) albumin (bromcresol green), urea (urease/glutamate dehydrogenase), creatinine (Jaffe reaction) were performed by Siemens Advia 1800 analyzer (Germany). Also, glycosylated haemoglobin (HbA1c) (HPLC method) was determined by Adams HA-8160 analyzer (Japan).

#### Urine samples

The 55 urine samples which were collected in a particular time interval were included in the study. The urine samples of subjects were collected into 75-mL sterile containers (Kayline Plastics, The Barton, South Australia, 5031). To control the urine concentration, data were normalized to urine creatinine concentration. Urinary creatinine was measured in spot urine samples by Dade Behring Dimension RXL autoanalyzer (Germany). Then, all urine specimens were stored frozen at −70°C until testing at the end of the collection period.

To minimize interbatch analytical variation, all samples from any volunteer were analysed in two batches for CAT, CuZnSOD and MDA; therefore, 30 different batches were run over a period of 30 days. The same lot of combined standard and quality control (QC) material were used throughout, and analyses were performed by a single analyst. Aliquots of a single urine pool, stored at −70°C, were used as the in-house QC material, and analysed in duplicate in each batch.

#### Determination of oxidative stress biomarkers in urine

Method of oxidative stress biomarkers in human blood were adopted in human urine (12, 13). At the start of our study, all urine samples were diluted with 1:50 serum physiologic (0.9% NaCI) for oxidative stress biomarkers analysis.

##### Superoxide dismutase assay

CuZnSOD activity in the urine samples was measured by the method of Fridovich [16]. The method for CuZnSOD activity employed xanthine and xanthine oxidase to generate superoxide radicals which react with p-iodonitrotetrazolium violet (INT) to form a red formazan dye which was measured at 505 nm. Assay medium consisted 0.01 M phosphate buffer, 3-cyclohexylamino-1-propanesulfonic acid (CAPS), buffer solution (50 mM CAPS, 0.94 mM EDTA, saturated NaOH) with a pH of 10.2, solution of substrate (0.05 mM xanthine, 0.025 mM INT), and 80 U/L xanthine oxidase. CuZnSOD activity was expressed as U/L.

##### Catalase assay

CAT activity in the urine samples was measured by the method of Beutler [17]. CAT activities were determined by measuring the decrease in hydrogen peroxide concentration at 230 nm. Assay medium consisted 1 M Tris–HCl, 5 mM Na_2_EDTA buffer solution (pH 8.0), 1 M phosphate buffer solution (pH 7.0), and 10 mM H_2_O_2_. CAT activity was expressed as U/L.

##### Malondialdehyde assay

MDA concentration, as an indicator of lipid peroxidation, in the urine samples were determined according to procedure of Ohkawa [18]. The reaction mixture contained 0.1 mL sample, 0.2 mL of 8.1% sodium dodecyl sulfate, 1.5 mL of 20% acetic acid, and 1.5 mL of 0.8% aqueous solution of thiobarbituric acid. The mixture pH was adjusted to 3.5 and the volume was finally made up to 4.0 mL with distilled water and 5.0 mL of the mixture of n-butanol and pyridine (15:1, v/v) was added. The mixture was shaken vigorously. After centrifugation at 4000 rpm for 10 minutes, the absorbance of the organic layer was measured at 532 nm. MDA concentration was expressed as nmol/mL.

### Glomerular filtration rate (GFR) assay

The GFR is considered most suitable for quantifying renal function. Practical limitations exist in measuring GFR directly, especially in acutely ill patients. Several reliable equations incorporating clinical variables to estimate the GFR are available; we used the Modification of Diet in Renal Disease equation [19].

### Statistical analysis

Seven urine samples were taken from each individual on the zero, 1st, 3rd, 5th, 7th, 15th and 30th days. Variation analyses were performed on natural logarithmic transformed data after exclusion of outliers using Cochran and Reed tests. The Cochran test did not highlight any outliers among duplicate measurements; however, it did identify results for seven samples from different subjects (five T2DM, all analytes; and two healthy individuals, MDA) as outliers among within-subject variances. The Reed test identified MDA for one healthy individual as an outlier. After exclusion of outliers, biological variation data were estimated according to the method published by Fraser et al. [20-24]. Analytical variance (SD_A_^2^) was calculated from the differences between the duplicates according to the formula:

$$ S{D_A}^2 = \varSigma {d}^2/2n $$

where d is the difference between duplicates and n is the number of duplicates. The SD_A_^2^ is expressed as relative SD_A_ to the first sample concentration, analytical coefficient of variation (CV_A_). For each analyte, one-way analysis of variance was used to divide the total variance into between-subject (SD_G_^2^) variance and total within-subject variance (SD_TI_^2^). Since SD_TI_^2^ includes both biological and analytical components, the within-subject variance (SD_I_^2^) was obtained by subtraction using the formula:

$$ {S_I}^2 = {S}_{TI}-{S_A}^2 $$

Within-subject and between-subject biological variations were expressed as the coefficient of variation by the use of homeostatic mean of each individual, within-subject coefficient of variation (CV_I_) and the overall mean, between-subject coefficient of variation (CV_G_), respectively. Index of individuality (II) was calculated as CV_I_/CV_G_ whereby a low value (<0.60: high individuality) indicated a low usefulness of population-based reference intervals, whereas a high value (>1.40: low individuality) indicated a high usefulness of population-based reference intervals, particularly when an unusual result was repeated for verification.

RCV, which is the difference required for two serial measurements of the oxidative stress biomarkers to have significantly changed at p < 0.05, was calculated as in [25]:

$$ RCV = {2}^{1/2}x\ Z\ x\ {\left(C{V_A}^2 + C{V_I}^2\right)}^{1/2} = 2.77C{V}_{TI} $$

Z-score was accepted as 1.96 (95% probability, bidirectional z-score).

Biological variation data for oxidative stress biomarkers were used to estimate the desirable quality specifications for I, B and TE, using the following formulas [26].

$$ I = 0.5C{V}_I $$

$$ B = 0.25{\left(C{V_I}^2 + C{V_G}^2\right)}^{1/2} $$

$$ TE = 1.65\left(0.25C{V}_I\right) + 0.25{\left(C{V_I}^2 + C{V_G}^2\right)}^{1/2} $$

Differences of the mean biological within-subject variances between the reference groups (DN/healthy, T2DM/healthy, DN/T2DM, as paired comparison) were checked by the *F* test. Linear regression analysis was used to check for significant trends in values for CAT, CuZnSOD and MDA and to investigate the time dependence of the within-subject variations. Group comparisons (like male versus female, DN/T2DM, DN/healthy, T2DM/healthy, etc.) were performed using the Mann–Whitney *U* test. A p value <0.05 was considered to be statistically significant.

## Results

The demographic characteristics and results of some biochemical analytes of the reference groups were listed in Table 1. The values of body mass index (BMI) were similar in all groups. HbA1c and FBG concentrations in patients with DN and T2DM were higher than healthy individuals. Serum creatinine concentrations in male were higher than female, and there was significant difference according to gender (p < 0.05). However, there was no significant difference according to age (p > 0.05). Estimated GFR in DN was seen at stage 3 of kidney damage.

Of the 1155 data points, 8 were classified as outliers. The number of remaining data points for each analyte is shown in Table 2. A uniform distribution of these outliers was observed among samples and subjects. The activities of CuZnSOD and CAT decreased, and MDA concentrations increased in patients with DN compared to healthy individuals (p < 0.05). However, all of them except CuZnSOD increased in patients with T2DM compared to healthy individuals (p < 0.05).

**Table 2:** **The levels of oxidative stress biomarkers in all of the reference groups**

	**Mean**	**Median**	**Reference interval**
T2DM group			
CAT	0.63*	0.62	0.25-0.95
CuZnSOD	5.08*	4.99	3.00-7.34
MDA	3.87*	3.92	1.00-6.98
DN group			
CAT	0.24*	0.23	0.12-0.37
CuZnSOD	3.68*	3.60	1.87-6.00
MDA	4.56*	5.01	1.09-7.00
Healthy Individuals			
CAT	0.36	0.35	0.15-0.73
CuZnSOD	5.39	5.08	2.77-10.70
MDA	0.59	0.54	0.10-1.53

While the results of CAT and CuZnSOD activities were expressed as U/L, MDA concentration was expressed as nmol/mL.

*Significant differences in patients with T2DM and DN compared to healthy individuals (p < 0.05).

Table 3 shows that the means of total within-subject variations in patients with DN and T2DM were significantly higher than healthy individuals (p < 0.05). Also, biological within-subject variances of MDA were larger than between-subject variances in all reference groups. However, within-subject variances of CAT and CuZnSOD were smaller than between-subject variances in all reference groups. While MDA showed low individuality (II > 1.40), CAT and CuZnSOD showed strong individuality (II < 0.60). The RCV values of CAT, CuZnSOD and MDA in patients with T2DM and DN were markedly higher than healthy individuals (p < 0.05).

**Table 3:** **Biological variation components in patients with T2DM or DN and healthy individuals**

	^**a**^ **CV**_**TI**_ **(%)**	^**b**^ **CV**_**G**_ **(%)**	^**c**^ **II**	^**d**^ **RCV (%)**
T2DM patients				
CAT	18.09	32.07	0.56	50.10
CuZnSOD	14.45	27.03	0.53	40.02
MDA	31.39	17.28	1.81	86.95
DN patients				
CAT	15.14	29.67	0.51	41.93
CuZnSOD	8.61	24.02	0.35	23.84
MDA	25.40	16.25	1.56	70.35
Healthy Individuals				
CAT	9.08	23.48	0.38	27.92
CuZnSOD	5.60	31.08	0.18	15.51
MDA	21.00	11.96	1.50	49.86

While the results of CAT and CuZnSOD activities were expressed as U/L, MDA concentration was expressed as nmol/mL.

^a^CV_TI_, within-subject coefficient of variation; ^b^CV_G_, between-subject coefficient of variation; ^c^II, index of individuality; ^d^RCV, reference change value (with 95% confidence).

Data regarding the imprecision of the method are presented in Table 4. The imprecision of our laboratory method for measuring CAT, CuZnSOD and MDA in urine was less than the desirable imprecision goals. For each analyte, the imprecision of the laboratory method and the desirable specifications for imprecision (I), bias (B), and total error (TE) derived from the biological variation data are presented in Table 5. The described methodology achieves these goals, with analytical CVs of < 3.5% for all analytes. Goals for bias and total error were 6.0-7.9% and 12.5-23.3%, respectively.

**Table 4:** **Precision (CV) of oxidative stress biomarkers’ measurements in urine using the described spectrophotometric method**

	*Within batch		**Between batch	
**Analyte**	**Mean**	**CV (%)**	**Mean**	**CV (%)**
CAT (U/L)	0.31	1.6	22.20	3.1
CuZnSOD (U/L)	5.19	2.3	4.99	3.3
MDA (nmol/mL)	0.57	1.9	0.49	3.2

*Within batch imprecision was calculated by analysing a pooled urine sample 10 times on the same day. **Between batch imprecision data were obtained from the in house QC material analysed in duplicate in 30 batches over a period of 30 days, as described in the statistical analysis section.

**Table 5:** **Laboratory method imprecision (CV**_**A**_ **) and desirable specifications for imprecision (I), bias (B) and total error (TE) derived from biological variation**

	**Desirable specifications**			**Method imprecision**
**Analytes**	**I (%)**	**B (%)**	**TE (%)**	**CV**_**A**_ **(%)**
CAT	4.5	6.3	13.7	3.1
CuZnSOD	2.8	7.9	12.5	3.2
MDA	10.5	6.0	23.3	3.3

The regression analysis showed no trends for the changes in the activities of CAT and CuZnSOD, and MDA concentration during 30 days in all reference groups.

## Discussion

To our knowledge, this is the first study carrying out the determination and application of data on biological variations of oxidative stress biomarkers in urines of patients with T2DM and DN as well as healthy individuals. Limited studies have been conducted to address these methodological issues and there is still controversy over which biomarkers to use. It has been suggested that oxidative stress biomarkers should be utilized for research purposes until more sensitive and specific assays are developed [27]. In this study we generated data of biological variations for oxidative stress biomarkers that are commonly measured in the laboratory when assessing oxidative stress.

In measuring the within-subject variation in chronic diseases one must define a time interval during which the process of the disease is practically stable and the consecutively measured individual values are independent of each other. Under these conditions, the measured within-subject variation can be used to derive decision-making criteria in long-term monitoring of the disease. During 30-days-intervals in this study the process of DN and T2DM could be regarded as stable. In our study, the biological within-subject variation of CuZnSOD, CAT and MDA analytes was statistically higher in the DN and T2DM subjects than in healthy individuals. The high glucose concentration in both T2DM and DN patients, as well as renal impairment in DN patients may be the cause of high within-subject variations. In spite of the fact that these individuals have a higher within-subject variation, we suggest that, for these the mean values should be used in deriving decision criteria. However, we found no significant difference in the within-subject variation of CAT and CuZnSOD in all groups, so these antioxidant enzymes can be used in deriving criteria for decision making. Furthermore, our results indicated that between-subject variations for CAT and CuZnSOD in all groups were generally larger than within-subject variation. We thought that CAT and CuZnSOD displaying small within-subject variation also allow more precise knowledge of the homeostatic set point and leave less margin for ambiguity in recognizing the patient’s status. However, our estimates of within-subject variations for MDA indicating that there were significant differences may be attributed day-to-day biological variation in lipid peroxidation. Also, we found that the mean total between-subject variances of MDA were smaller than within-subject variances in all groups, probably because of the lack of homogeneity in the within-subject variation and perhaps due to problems of stability. The lack of homogeneity in the data of MDA analyte may cause erroneous calculations of biological variation and false interpretations of results, as such calculations involve an analysis of means and may incorporate individuals with within-subject variation values much larger than between-subject variations. The II has been used by many to investigate the utility of conventional population-based reference values. For a high index of individuality, >1.4, it has been reported that reference intervals will be more useful than for a low index, <0.6. For quantities with very low indices, a repeat test result will be close to the first and will provide no new information, whereas for quantities with high indices, a repeat test will decrease the number of true and false positivity [28,29]. In our study, the calculated indices of individuality for CAT and CuZnSOD in T2DM, DN and healthy subjects in all groups were less than 0.6 which showed conventional reference values to be of little utility for interpretation. Thus CAT and CuZnSOD examined in this study will be of little use in the diagnosis of early or latent of diabetes. The results of our studies were similar to results of Covas et al. [30]. This author reported that II for CuZnSOD and glutathione peroxidase in blood was 0.45, suggesting that it has little value as a diagnostic or screening tool. In our study, the II for MDA was found to be >1.4 according to the discriminant values proposed by Fraser and Harris [20]; hence this analyte may be useful for diagnosis and screening of T2DM or DN. At this point, it would be worthwhile to check for statistically significant differences according to sex, to see whether a separation of reference values is indicated.

The RCV is an important clinical tool for the assessment of changes in patients being monitored in pathological situations [25]. The present study showed that II < 0.6 indicates the range at which RCVs for CAT and CuZnSOD may be substituted for the reference interval. However, RCVs for CAT and CuZnSOD were higher in patient groups than healthy individuals. Hence, RCVs for CAT and CuZnSOD may be clinically useful in T2DM and DN. Therefore, the RCV for predicting crises in urine of both diabetic groups represents an optimization of laboratory reporting and may be a valuable tool for clinical decision making. Fraser et al. [31] reported that RCV can be used to set objective criteria for use in delta-checking quality control techniques. Rather than spending considerable resources in defining reference intervals, laboratories are urged to apply well-established methodology to calculate RCV and to use these in everyday practice, providing considerable advantage in the monitoring of changes in serial results from individuals.

The current European consensus is that analytical quality specifications are best based on components of biological variation [26]. We have, as far as we are aware, documented the biological variation of oxidative stress in urine for the first time. In our study, a desirable analytical CV of 0.5 times CV_I_ is usually considered because this assay imprecision adds only a 23% to the data variability [32]. In the present study, the imprecision of our laboratory methods for measuring oxidative stress biomarkers in urine was found less than the desirable imprecision goals. Analytical quality specifications were derived from biological variation data, and imprecision goals can be reasonably achieved with current methods. Therefore, it is hoped that these biological variation data may serve to apply this approach in the analysis of urinary oxidative stress biomarkers.
